# Effective treatment with icotinib in advanced lung adenocarcinoma harboring rare *EGFR* mutation G719A/L833V: A case report

**DOI:** 10.1097/MD.0000000000030080

**Published:** 2022-08-19

**Authors:** Bin Zhou, Yanan Wang, Haijiang Liao, Ben Li

**Affiliations:** a Department of Thoracic Surgery, Affiliated Hospital of Hebei University, Baoding, China; b Department of Pathology, Affiliated Hospital of Hebei University, Baoding, China.

**Keywords:** case report, double mutation, *EGFR* G719A/L833V, icotinib, non–small cell lung cancer

## Abstract

**Rationale:**

Mutations in *epidermal growth factor receptor* (*EGFR*) play critical roles in the pathogenesis of non–small cell lung cancer (NSCLC), and they are highly associated with sensitivity to tyrosine kinase inhibitors. Targeted therapies are approved for patients with “classical” mutations and a small number of other mutations. However, patients with rare, even double *EGFR* mutations have different responses to EGFR tyrosine kinase inhibitor, which brings uncertainty to clinical practice.

**Patient concerns:**

A 74-year-old woman, never-smoker, was presented with chest pain. Chest computed tomography scan showed a big lesion in the right upper lobe with mediastinal lymph nodes metastases. Fine-needle biopsy and pathology suggested lung adenocarcinoma. A rare G719A/L833V double mutation of *EGFR* was detected in both tissue and plasma samples by next-generation sequencing.

Interventions and outcomes:

Icotinib was used as first-line therapy and showed good efficacy. Partial response was achieved, and the progression-free survival was 8 months.

**Lessons:**

This is the first report of the icotinib treatment achieving long-lasting and stable disease control in an NSCLC patient with *EGFR* G719A/L833V mutation. Icotinib could be a first-line treatment option in NSCLC patients harboring *EGFR* G719A/L833V mutation.

## 1. Introduction

Lung cancer is the most common cause of cancer death worldwide, with an estimated 1.8 million deaths each year, and adenocarcinoma is the most common histological type of lung cancer.^[1]^ *Epidermal growth factor receptor* (*EGFR*) gene mutation is detected in 30% to 40% of lung adenocarcinoma and is associated with specific demographic parameters such as never-smoker, Asian, and female gender.^[2,3]^ Mutations such as *EGFR* exon 19 deletion (19del) and L858R, which are termed “classical mutations,” comprise approximately 90% of all *EGFR* mutations. Patients with classical mutations show marked improvements in clinical outcomes when treated with first-, second-, or third-generation tyrosine kinase inhibitors (TKIs).^[4,5]^ The first-generation EGFR TKIs include erlotinib, gefitinib, and icotinib; the second-generation TKIs include afatinib and dacomitinib; and the third-generation TKIs include osimertinib and almonertinib. Other *EGFR* mutations in the kinase domain (exons 18−21) have also been established as oncogenic drivers of non–small cell lung cancer (NSCLC) and confer different sensitivity or resistance to EGFR TKIs.^[6]^ Patients with uncommon *EGFR* mutations show heterogeneity. The effects of EGFR TKIs on patients with rare *EGFR* mutations are still largely unknown. There is an urgent need to determine the clinical significance of uncommon mutations in *EGFR*, particularly their sensitivity to TKIs.

The G719X mutation in *EGFR* refers to point mutations that result in substitutions of the glycine at position 719 to other residues, primarily alanine (G719A), cysteine (G719C), and serine (G719S). The G719X mutation accounts for approximately 3% of all *EGFR* mutations in Asian and Caucasian populations.^[7]^ However, double mutations are scarcer, and their sensitivities to TKIs are more obscure than the G719X single mutation. The *EGFR* L833V mutation was mostly reported to be accompanied with H835L with or without other mutations in only 11 NSCLC patients reported by now.^[8,9]^ Here, we report a patient with stage III lung adenocarcinoma harboring rare *EGFR* G719A/L833V mutations, and a beneficial clinical effect was observed after icotinib treatment.

## 2. Case presentation

**Figure 1.**
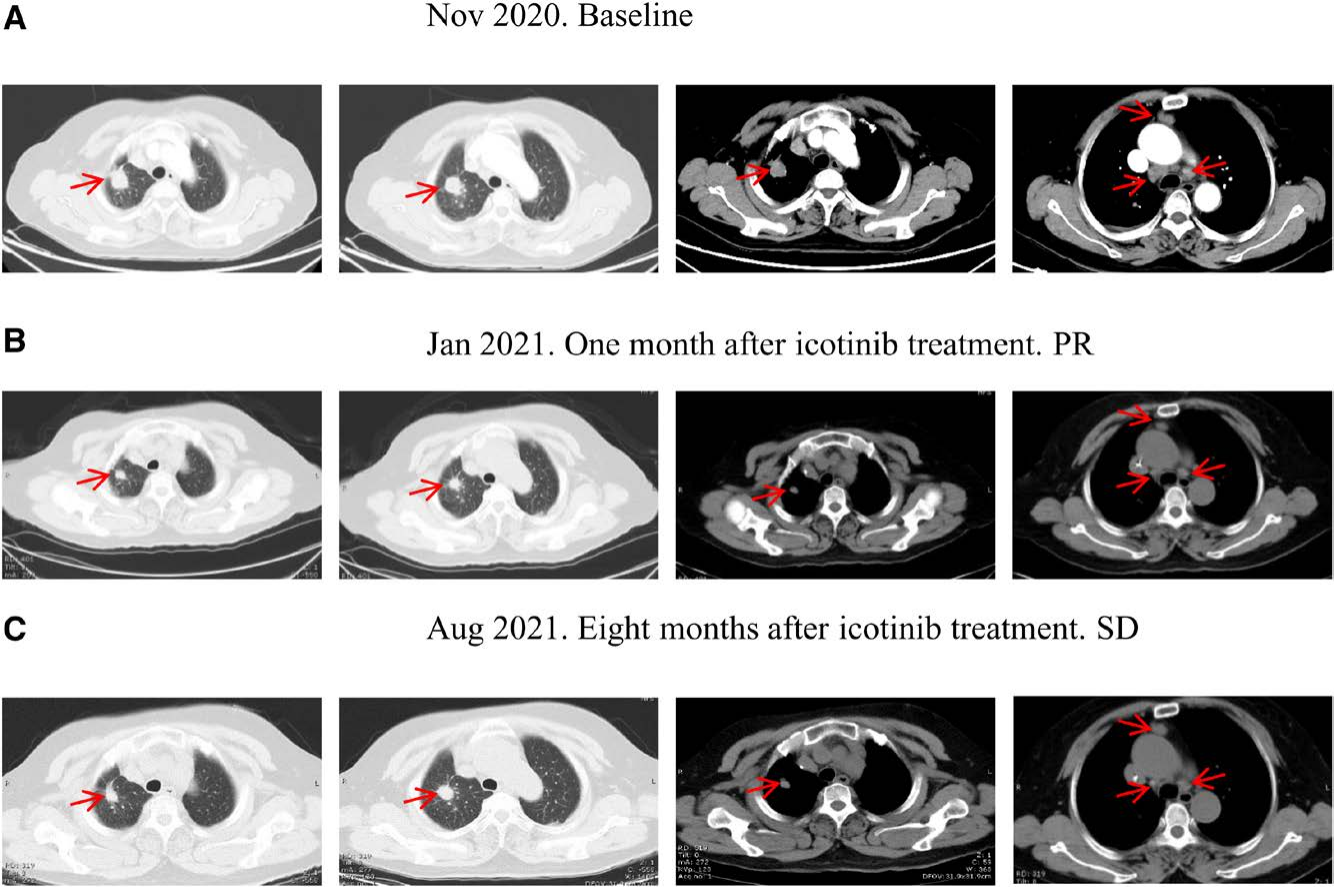
Primary lung cancer and mediastinal lymph nodes metastases before and after icotinib therapy. The red arrows indicate the tumor lesions. PR = partial response, SD = stable disease.

A 74-year-old woman, never-smoker, was presented with chest pain for 4 days in November 2020. The patient had a personal history of transient ischemic attack and a history of hepatic hemangioma and coronary heart disease before admission. On admission, her general condition was normal. Her heart rate was slow (43 beats/min). Chest auscultation demonstrated diminished breath sounds in the right lung field. Chest computed tomography scan showed a 2.5 cm × 1.8 cm mass lesion in the right upper lobe with lobulation, spiculation and vascular convergence, multiple enlarged mediastinal lymph nodes (the largest one 2.2 cm × 1.3 cm), and pleural effusions, suggesting peripheral lung cancer with mediastinal lymph nodes metastases (Fig. 1A).

**Figure 2.**
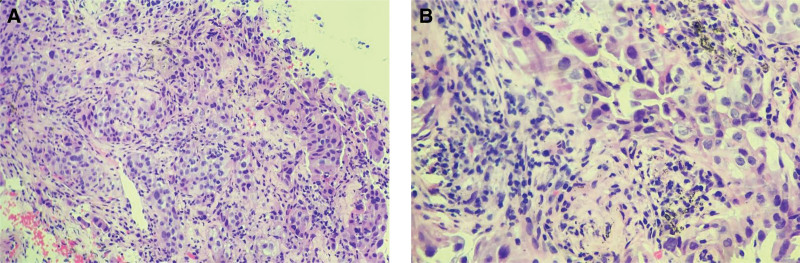
H&E staining of lung adenocarcinoma. (A) H&E staining 200×. (B) H&E staining 400×. H&E staining of the patient’s biopsy and the tumor cells were characterized by the variation in nucleus size and shape, the deep staining, and the increased nucleoplasm index, which was marked by a closed curve. H&E = hematoxylin and eosin.

Diagnoses:

The patient underwent a fine-needle biopsy of the right upper lobe. Immunohistochemically, tumor cells in the main part were CK7 (+), TTF1 (+), Napsin A (+), P40 (–), and CK5/6 (+). She was diagnosed with stage III lung adenocarcinoma (Fig. 2). A combination of pemetrexed disodium and carboplatin was administered. After 1 month’s treatment, the patient achieved a stable disease.

To find a candidate for target therapy, we performed capture-based next-generation sequencing with a panel of 168 cancer-related genes (Lung Plasma, Burning Rock, Guangzhou, China) with lung biopsy specimens and whole blood cells. *EGFR* c.2156G>C (p.G719A) and c.2497T>G (p.L833V) mutations were identified in both tissue and plasma circulating tumor DNA. Subsequently, the patient initiated icotinib (125 mg tid) treatment in December 2020. The patient achieved a partial response (PR) 1 month later (Fig. 1B) with the shrunken right upper lesion (2.2 cm × 1.2 cm) and lymph nodes (the largest one 1.0 cm). She experienced an overall improvement in her clinical symptoms, and PR was confirmed after 2 months of treatment, with the lesion measuring 1.4 cm × 1.2 cm. The patient achieved durable clinical benefit with a progression-free survival (PFS) of 8 months (Fig. 1C). There was no significant side effect during icotinib treatment. A computed tomography scan demonstrated an enlarged lung lesion (1.8 cm × 1.5 cm) and lymph nodes on the most recent follow-up in August 2021. After disease progression, she’s taking a comprehensive examination for further treatment. Written informed consent was obtained from the patient for publication of this case report and any potentially identifying information and images.

## 3. Discussion

In this case, a 74-year-old woman, never-smoker, was diagnosed with clinical stage III lung adenocarcinoma. Next-generation sequencing identified 2 uncommon *EGFR* mutations, G719A and L833V, from tissue and circulating tumor DNA. After icotinib treatment, multiple nodular lesions continued to be alleviated, and PR was achieved after only 1 month’s treatment, suggesting that icotinib has a therapeutic effect on NSCLC with *EGFR* G719A/L833V compound mutation. The patient had a PFS of 8 months with icotinib treatment. This is the first case of first-line treatment with icotinib in an NSCLC patient harboring *EGFR* G719A/L833V mutation.

There are no clearly established guidelines for EGFR TKIs treatment for patients with uncommon *EGFR* mutations. The heterogeneous sensitivity to EGFR TKIs of uncommon *EGFR* mutations should be considered when making a clinical decision. Double mutations of G719A and L833V in *EGFR* rarely coexist in lung cancer, and few published studies have detailed the clinical profiles. Only 1 patient with double mutations of G719A and L833V has been reported.^[10]^ The patient was treated with gefitinib, and the best response was PD with a PFS of only 2 months. The G719X mutation showed intermediate sensitivity to first-generation TKIs, like gefitinib and erlotinib.^[11,12]^ Previous work also showed that the second generation of EGFR TKIs, like neratinib and afatinib, effectively treat lung adenocarcinoma with the *EGFR* G719X point mutation.^[13,14]^

Chen et al^[15]^ showed that *EGFR* mutations are seldom singlets but are almost doublets if the mutations occur at 1 of the 5 amino acids, E709, G719, S768, T790, and L861.^[15]^ They also found that *EGFR* doublets have enhanced oncogenic potential, resulting in the frequent recurrence of such doublet pairs. Our case is consistent with Chen’s study. L833V mutation was seldom reported as singlets and was mostly found with H835L and showed clinical response to the first and third-generation TKIs.^[16–18]^

A clinical challenge for physicians treating patients with *EGFR*-mutant cancers is to appropriately identify and match patient mutations with the best EGFR TKIs. Icotinib is an orally active, first-generation EGFR TKI, one of the standard drugs for treating advanced NSCLC in China.^[19]^ Our case showed that patient with *EGFR* G719A/L833V double mutation is more likely to benefit from icotinib treatment. However, more evidence is needed for the clinical significance of icotinib in patients with rare *EGFR* mutations, especially complex mutations. Our case also has other limitations. The patient was lost to follow-up after disease progression with icotinib because of the coronavirus disease 2019 pandemic and we failed to assess her overall survival.

## 4. Conclusion

This is the first report of the icotinib treatment achieving long-lasting and stable disease control in an NSCLC patient with *EGFR* G719A/L833V mutation. Our results will help guide the use of EGFR TKIs in patients with uncommon *EGFR* mutations.

## Acknowledgments

The authors would like to thank Dr. Ying Sun and Dr. Chunxiao Pan from Burning Rock Biotech for their suggestions in data analysis and manuscript writing.

## Author contributions

BZ: wrote the manuscript and collected data. HL: collected and processed data. YW: reviewed and edited the paper. BL: designed the study and reviewed the paper. All authors read and approved the final manuscript.

**Conceptualization:** Haijiang Liao, Yanan Wang.

**Data curation:** Bin Zhou.

**Funding acquisition:** Ben Li, Yanan Wang.

**Project administration:** Haijiang Lia.

**Supervision:** Ben Li, Yanan Wang.

**Validation:** Ben Li.

**Writing – original draft:** Ben Li, Bin Zhou, Haijiang Lia.

**Writing – review & editing:** Ben Li.
